# Implementation of a Stress Biomarker and Development of a Deep Neural Network-Based Multi-Mental State Classification Model

**DOI:** 10.3390/bioengineering12121352

**Published:** 2025-12-11

**Authors:** Sangsik Lee, Jaehyun Jo, Sohyeon Bang, Jinhyoung Jeong

**Affiliations:** 1 Department of Digital Healthcare, Catholic Kwandong University, 24 Beomil-ro 579 Beongil, Gangneung-si 25601, Republic of Korea; lsskyj@cku.ac.kr (S.L.); jh_507@cku.ac.kr (J.J.); 2 Department of Electronic and Communication Engineering, Catholic Kwandong University, 24 Beomil-ro 579 Beongil, Gangneung-si 25601, Republic of Korea; thgus7572@cku.ac.kr; 3 Department of Healthcare Management, Catholic Kwandong University, 24 Beomil-ro 579 Beongil, Gangneung-si 25601, Republic of Korea

**Keywords:** stress prediction, biomarker, mental health multi-classification, physiological signals, transformer model

## Abstract

The purpose of this study was to develop a model capable of predicting stress levels and interpreting the underlying physiological patterns using large-scale, real-life biosignal data. To achieve this, we utilized approximately 137,000 longitudinal measurements voluntarily collected from residents of Sejong Special Self-Governing City over a two-year period (February 2023–December 2024). Based on these data, we constructed a stress prediction framework that integrates both static machine-learning models—such as Random Forest and LightGBM—and time-series deep learning models, including LSTM and Transformer architectures. Model interpretability was further enhanced through SHapley Additive exPlanations (SHAP), which quantified the contribution of key biomarkers, and through visualization of Transformer attention weights to reveal temporal interactions within the biosignal sequences. The central objective of this study was to evaluate how accurately a deep learning model can learn and reproduce stress indices generated by existing heart rate variability (HRV)-based algorithms embedded in K-FDA-approved wearable devices. Accordingly, the ground truth used in this work reflects algorithmic outputs rather than clinically validated assessments such as salivary cortisol or psychological scales. Thus, rather than identifying independent clinical stress markers, the present work focuses on determining whether a Transformer-based model can effectively approximate device-derived physiological stress levels over time, thereby providing a methodological foundation for future applications using clinically validated stress labels. Experimental results demonstrated that the Transformer model achieved approximately 98% classification accuracy across this large dataset, indicating that it successfully captures short-term biosignal fluctuations as well as long-term temporal structure. These findings collectively demonstrate the engineering feasibility of developing a large-scale, wearable-based stress monitoring system.

## 1. Introduction

Stress is widely recognized as a factor that significantly impacts human health and social well-being. Chronic or excessive stress can disrupt the body’s homeostatic balance, leading to the onset or exacerbation of various physical and psychological disorders, and is increasingly regarded as a major public health concern [1,2,3]. Accordingly, the ability to detect or predict stress in real time has become an important research focus, with the potential to support timely interventions and personalized stress management strategies that prevent stress-related complications [4,5].

Over the past decade, studies utilizing physiological signals for stress prediction have increased substantially, supported by the rapid adoption of wearable technologies. Recent research has primarily relied on heart rate variability (HRV), electrodermal activity (EDA), respiration, and photoplethysmography (PPG) as key biomarkers because these modalities exhibit characteristic autonomic changes under stress conditions [6,7,8]. Traditional machine learning classifiers—such as SVM, random forests, and k-nearest neighbors—have been widely applied to these biosignals and have demonstrated promising performance, particularly in laboratory-induced stress environments [7,9]. However, despite these advances, such approaches often depend on handcrafted features and fail to fully capture the temporal complexity of physiological responses.

More recently, deep learning models capable of modeling sequential biosignal patterns have gained attention. Recurrent neural networks (RNNs), long short-term memory (LSTM) networks, and temporal convolutional networks (TCNs) have shown strong performance in learning both short-term and long-term physiological dynamics [10,11]. Attention-based architectures, particularly Transformer models, have further advanced time-series analysis by providing the ability to highlight salient moments or signal dimensions associated with stress [12]. These models have demonstrated enhanced predictive accuracy and offer a mechanism for interpreting which temporal patterns contribute most to classification outcomes, addressing a key limitation of earlier black-box approaches.

Despite these developments, existing literature still presents notable limitations. Many studies rely on small sample sizes or controlled laboratory datasets, limiting generalizability to real-world conditions. Furthermore, the explainability of stress prediction models remains insufficient in practice, even as explainable AI (XAI) techniques—such as SHAP—gain traction in broader biomedical modeling [13]. Very few studies integrate both high-performance temporal modeling and systematic interpretability analysis with respect to well-established physiological stress markers.

Stress differs from many other biomedical prediction targets in that it is a highly subjective and multifaceted psychophysiological response [14]. As such, users must be able to understand and trust the basis of the model’s predictions, which makes interpretability particularly essential in this domain. Physiological-signal–based stress prediction relies on complex and dynamic biomarkers—such as HRV, heart rate fluctuations, and autonomic nervous system activity—making it critical to identify which specific features drive a model’s decision. This underscores the importance of adopting SHAP (Shapley Additive Explanations) to overcome the inherent “black-box” nature of deep learning models. Furthermore, analysis of real-world data from residents of Sejong Special Self-Governing City revealed substantial individual variability in physiological stress responses: even under similar conditions, users exhibited personalized autonomic and HRV patterns [15]. SHAP-based interpretation enables individualized explanations by highlighting the features that most strongly contribute to each prediction, thereby enhancing transparency and reliability in both clinical and daily life stress monitoring.

Building on these gaps, the present study proposes a Transformer-based deep learning framework for multivariate biosignal time-series data with an emphasis on both predictive accuracy and physiological interpretability. The model leverages autonomic and vascular biomarkers to capture complementary aspects of the stress response and incorporates SHAP-based feature attribution and attention-based visualization to identify physiologically meaningful contributors to prediction outcomes. Through this approach, the study aims to overcome the interpretability and generalizability limitations observed in previous work and provide a foundation for more reliable, transparent, and personalized stress monitoring.

However, it should be clarified that the “stress level” defined in this study is not a clinical diagnosis established by medical professionals, but rather a quantitative indicator generated by the HRV- and autonomic balance–based algorithm embedded in the wearable device. Consequently, the modeling in this study does not aim to establish a new medical or causal relationship between biosignals and psychological stress. Instead, it focuses on evaluating how accurately a Transformer-based deep learning model can learn and replicate the device-derived stress indices in a time-series context. As wearable-based stress management becomes increasingly important, limitations of prior research—particularly the reliance on small-scale, laboratory-controlled datasets—underscore the need for methodological validation using large-scale real-life data. By leveraging real-world biosignal measurements collected from the general population, this study serves as a proof-of-concept that demonstrates the technical feasibility of using deep learning to approximate existing rule-based physiological algorithms. This framework provides an important foundation for future applications once clinically validated stress labels or medical gold-standard data become available.

## 2. Materials and Methods

### 2.1. Data Source and Composition

This study utilized a public stress biomarker dataset provided by AI Hub and originally collected through the “Smart Health Station” program operated by Sejong Special Self-Governing City, South Korea. The Smart Health Station is an unmanned community health monitoring booth that enables residents to voluntarily measure their physiological status using clinical-grade biosignal devices. The data collection period spanned approximately two years, from February 2023 to December 2024, thereby capturing seasonal fluctuations and long-term biometric variability across the population.

A total of 137,686 valid measurements after exclusion of missing or invalid values were used in the final analysis. These measurements were acquired from a broad adult demographic, with participants authenticating via a mobile application and performing self-measurements following standardized on-screen guidance. The dataset includes demographic variables, autonomic nervous system indices, vascular and fatigue-related indicators, and device-generated stress-level annotations.

All physiological features utilized in this study originate from raw photoplethysmography (PPG) signals collected by a clinical-grade pulse wave analyzer (PPG Analyzer, Max Pulse, Medicore, Seongnam-si, Republic of Korea), which is certified as a Class II medical device by the Korea Food and Drug Safety Agency (K-FDA). The stress assessment algorithm embedded in this device has undergone regulatory review and has been clinically validated for its correlation with standard HRV markers such as SDNN and RMSSD, as well as autonomic nervous system activity. Therefore, the stress labels used as ground truth in this study are not arbitrary manufacturer-defined outputs, but medically meaningful indicators with documented regulatory validation.

The raw PPG signals were processed entirely by the data provider before public release. HRV features were computed using standardized time- and frequency-domain analysis methods consistent with clinical HRV guidelines, while vascular indicators were derived from pulse waveform morphology using validated algorithms. Detailed acquisition parameters—such as device sampling frequency, sensor specifications, or internal filtering pipelines—are not disclosed to end users; however, the dataset was released only after internal verification and quality control by the data-generating organization.

To ensure signal reliability, the provider applied routine artifact-removal procedures, including exclusion of physiologically implausible values, removal of measurements affected by motion artifacts or sensor failure, and internal validation of derived indices. Only complete and high-quality physiological measurements were retained in the final dataset. Because the dataset was fully anonymized prior to distribution, with all personal identifiers removed, the present study was exempt from Institutional Review Board (IRB) review under the relevant provisions of the Korean Bioethics and Safety Act.

### 2.2. Data Preprocessing

Before model development, the raw dataset underwent a structured preprocessing pipeline to ensure analytical suitability and data quality. Missing values were present in several fields: the “administrative district” variable exhibited the highest missing rate (5061 records, 24.2%), whereas “gender” and “year of birth” contained only a single missing entry each (<0.01%). To handle these differences appropriately, all missing entries in the district variable were encoded as a separate “Unknown” category, while the isolated missing values in gender and birth year were imputed using the mode (female) and the mean birth year, respectively. Measurement date and time were merged into a single datetime variable, and each participant’s age at the time of measurement was computed and added as a new feature. Categorical variables, including gender and administrative district, were transformed using one-hot encoding, and continuous physiological indicators were standardized using z-score normalization (mean = 0, SD = 1) to reduce scale variance and ensure consistent contribution across features.

In addition to tabular preprocessing, standard physiological signal preprocessing procedures described in the dataset documentation were applied by the data provider prior to release. These included band-pass filtering to suppress high-frequency noise and motion artifacts, sliding-window segmentation to extract temporal HRV-related features, and removal of physiologically implausible outliers. All processed features were subsequently normalized on a per-subject basis to reduce inter-individual variability. The final dataset, consisting only of valid and fully processed measurements, was used for downstream model training.

### 2.3. Modeling Approach

This study developed two types of predictive models for stress-level classification: a static model and a time-series model. The static model treats each measurement as an in-dependent sample and uses only single-time-point features—including demographic at-tributes (gender, age, and administrative district) and physiological indicators such as heart rate, autonomic balance indices, fatigue, and cardiac stability. These multivariate features were input into ensemble tree-based classifiers (Random Forest and LightGBM) to directly predict the corresponding stress level (1–5) without incorporating temporal context.

In contrast, the time-series model captures temporal dynamics by constructing sequential inputs from repeated measurements recorded from the same user. A fixed-length window of W = 5 consecutive measurements was used to form each sequence. The temporal interval between adjacent measurements varies across individuals, with an average spacing of approximately three days, meaning that each sequence represents roughly two weeks of stress-related physiological fluctuations. These sequences were used to train deep learning architectures—specifically LSTM and Transformer models—that learn temporal dependencies and evolving autonomic patterns across the five consecutive time steps. The LSTM captures short- and long-term sequential structure, whereas the Trans-former leverages self-attention to model global temporal relationships across the entire sequence.

Although both modeling approaches use the same feature set and predict the same five-level stress label, the static model relies solely on individual observations, while the time-series model explicitly incorporates longitudinal information. As shown in our experimental results, models that exploit temporal structure (LSTM/Transformer) outperform static models by leveraging sequential physiological trends rather than isolated measurements.

This study employed both classification and regression frameworks because the dataset provides two complementary target variables: a five-level categorical stress stage and a continuous stress score. The classification model predicts the discrete stress level (1–5), which is intuitive for clinical or user-facing interpretation, whereas the regression model predicts the continuous stress index, allowing detection of subtle variations that are not captured by discrete classes. Using both approaches provides complementary benefits—interpretability through categorical outputs and fine-grained sensitivity through continuous estimates. Accordingly, classification metrics (accuracy, precision, recall, F1-score, ROC-AUC, PR-AUC) and regression metrics (MAE, RMSE, R^2^) were evaluated separately.

Table 1 summarizes the key hyperparameters and final selected settings of the Transformer model trained based on stress measurement data from Sejong Special Self-Governing City. It includes model structure-related parameters such as the number of Transformer encoder layers, the number of self-attention heads, and the embedding dimension, as well as optimization-related hyperparameters (learning rate, batch size, etc.).

Hyperparameter tuning was performed by utilizing a validation set to find the settings that maximized the model’s generalization performance. Approximately 20% of the total data was held out for validation, not used during training, and the loss (MSE) on the validation set was compared for various hyperparameter combinations. To simultaneously optimize multiple hyperparameters, an automated search technique was introduced. Specifically, the Optuna library, based on random search, was utilized. Unlike grid search, random search is a technique that randomly selects combinations within a specified range rather than trying all predefined combinations. In this experiment, appropriate search ranges were set for each hyperparameter. For example, the number of encoder layers was set to [2, 4, 6], the number of heads was set to [4, 8, 16], the embedding dimension was set to [64, 128, 256], and the initial learning rate was set to [0.0005, 0.001, 0.005]. Approximately 30 random trials were performed. Optuna’s Bayesian optimization function allowed us to explore promising areas further by incorporating the results of previous trials, efficiently finding the optimal combination even within a limited number of trials. During the exploration process, validation losses were recorded and compared for each model training session, ultimately selecting the hyperparameter set that achieved the lowest validation loss. The key findings from this tuning process are summarized below.

First, parameters determining model complexity (e.g., number of encoder layers, number of heads, embedding size) maximized performance at appropriate levels. Too low values resulted in the model failing to capture the complex patterns of the stress time series, resulting in high error rates. Conversely, excessively high values resulted in overfitting, with the training data exhibiting near-zero error rates but increasing validation errors. In fact, a model with 4 layers, 8 heads, and 128 embeddings achieved the lowest validation MSE, and deviating from these settings resulted in poor performance. This suggests the importance of maintaining a balance between adequate model capacity and regularization, as previously mentioned.

Second, adjusting the learning rate and optimization parameters significantly impacted model convergence and final performance. If the initial learning rate was too large, learning became unstable in training, while if it was too small, learning stalled before reaching the optimum. Tuning revealed that values around 0.001 yielded the best results, and models with a learning rate decay schedule achieved lower validation error than models without. This can be attributed to fine-tuning by lowering the learning rate later in training. Furthermore, the momentum effect of the Adam optimizer allowed for faster loss reduction compared to SGD.

Finally, the impact of dropout and regularization is noteworthy. Applying dropout consistently improved performance on the validation set, significantly reducing the final MSE compared to the unused setting. A dropout rate of 0.1 randomly removes the activations of some neurons during training, preventing the model from relying excessively on a few features. This contributes to improved generalization performance, especially in situations with limited training data. Conversely, increasing the dropout rate to 0.2 resulted in a slight decrease in performance due to a decrease in effective learning capacity, maintaining the value at 0.1. In summary, through systematic hyperparameter tuning, the Transformer-based stress prediction model in this study achieved an optimal structure (4-layer encoder, 8-head attention, etc.) and learning settings (Adam, learning rate 0.001 + decay schedule, etc.). These settings significantly reduced the model prediction error based on the validation data, significantly lowering the MSE compared to the initial settings before tuning, and simultaneously achieving a balance between model complexity and generalization performance. Therefore, the selected hyperparameter combination was optimized for the corresponding stress time-series data, contributing to the model’s effective prediction of an individual’s physical/mental stress index.

In time series modeling, continuous measurements from the same user are organized into a chronological sequence as input, and these temporal dependencies are learned to predict current or future stress levels. For example, LSTM and Transformer models sequentially accept a series of past measurements of a user and predict stress levels or scores. In this process, as with static models, data is partitioned to prevent duplication between the training and evaluation stages for the same individual. Training/validation sets are organized according to chronological order to evaluate future time-point prediction performance. Through the parallel use of static and time-series models, we aimed to compare and integrate approaches based on individual time-point characteristics with approaches analyzing patterns over time.

### 2.4. Performance Evaluation Indicators

The performance of the models was evaluated using metrics appropriate for both classification and regression tasks because two complementary target variables were provided in the dataset: a five-level categorical stress stage and a continuous stress score. The classification model predicts discrete stress levels (1–5), which are intuitive for clinical or user-facing interpretation, whereas the regression model predicts a continuous stress index that captures subtle variations not reflected in categorical labels. Using both modeling strategies enables complementary benefits—interpretability through discrete classes and fine-grained sensitivity through continuous estimates.

For the classification task, performance was evaluated using accuracy, precision, recall, and F1-score to assess balanced classification capability, along with ROC-AUC and PR-AUC to measure discriminative performance under potential class imbalance. In addition, a confusion matrix was used to analyze misclassification patterns across the five stress levels.

For the regression task, model accuracy was assessed using the coefficient of determination (R^2^), root mean squared error (RMSE), and mean absolute error (MAE), which quantify linear agreement and average prediction error. By jointly examining these classification and regression metrics, the predictive performance and error characteristics of each modeling approach were evaluated from multiple perspectives.

### 2.5. Model Analysis Techniques

To interpret the contribution of each feature to the model’s predictions, we applied SHAP (Shapley Additive exPlanations). For tree-based static models (Random Forest and LightGBM), TreeSHAP was used via the shap.TreeExplainer, which provides exact Shapley values by exploiting the tree structure.

For the Transformer-based time-series model, which is not compatible with TreeSHAP, a model-agnostic Shapley approximation was adopted. Specifically, a KernelSHAP-style perturbation approach was used, in which input features are masked or permuted to estimate their marginal contribution to prediction changes. This enables computation of Shapley attributions even for non-linear deep architectures.

SHAP values were first computed at the individual-sample level for all users, after which global feature importance was obtained by aggregating Shapley values across all samples. Summary plots were generated using the distribution of Shapley values per feature, where the x-axis reflects the direction and magnitude of each feature’s contribution, and color encodes the relative feature value. This global explanation provides an interpretable overview of which physiological or demographic variables most strongly influenced the model’s stress predictions.

**Figure 1:**
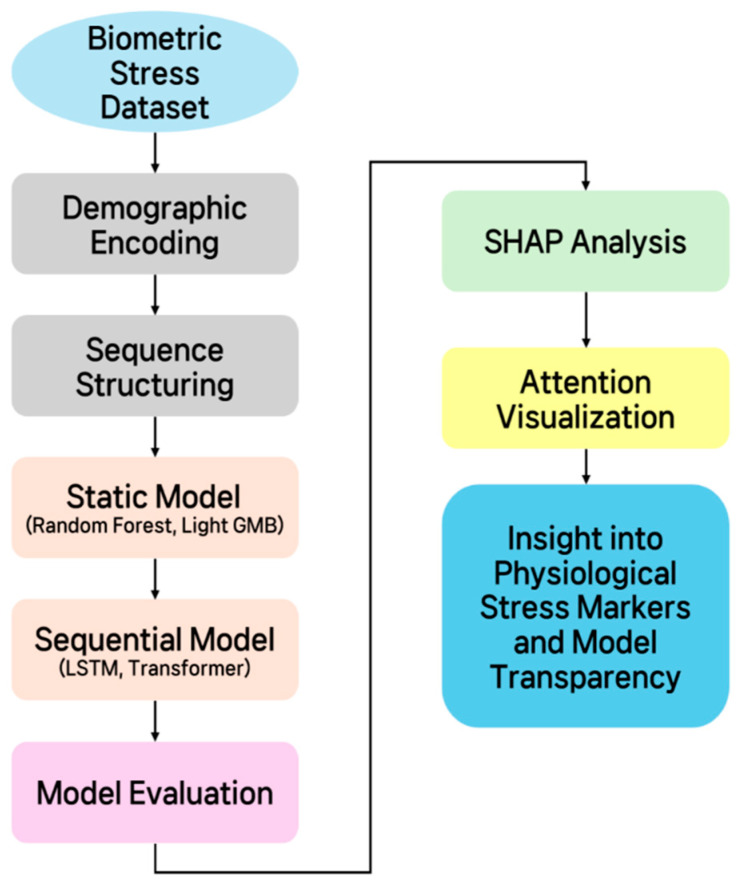
Schematic diagram of the overall methodology.

Figure 1 schematically illustrates the overall methodology proposed in this study. It outlines the process step-by-step, beginning with data organization and preprocessing, followed by defining the stress classification/regression problem, building static and time-series models, validating model performance using performance evaluation metrics, and finally, model interpretation. The diagram, designed to provide a comprehensive overview of the study’s analytical procedures, provides a clear overview of the key elements and processing flow at each stage.

## 3. Results

### 3.1. Model Performance Evaluation

#### 3.1.1. Classification Model Results

Because classification and regression were trained as two independent predictive tasks, Table 2 reports performance metrics for both task-specific models. In this subsection, we describe only the classification results.

The stress-stage classification performance of each model was evaluated using group-based cross-validation, and the summary of classification metrics is shown in Table 2. Among all models, the Transformer achieved the highest classification performance, with an accuracy of approximately 98%, followed by the LSTM model, which recorded an accuracy of around 97%. The Random Forest and LightGBM models achieved accuracies of approximately 94%, performing slightly lower than the deep learning models but still demonstrating strong predictive capability as baseline classifiers.

The macro-average F1-score, which reflects balanced performance across the five stress levels, was also highest for the Transformer model, slightly surpassing LightGBM. This indicates that the Transformer not only achieved high overall accuracy but also maintained stable performance even for relatively low-frequency classes, whereas LightGBM—although showing strong overall accuracy—exhibited mild imbalance in some intermediate stress levels.

**Figure 2:**
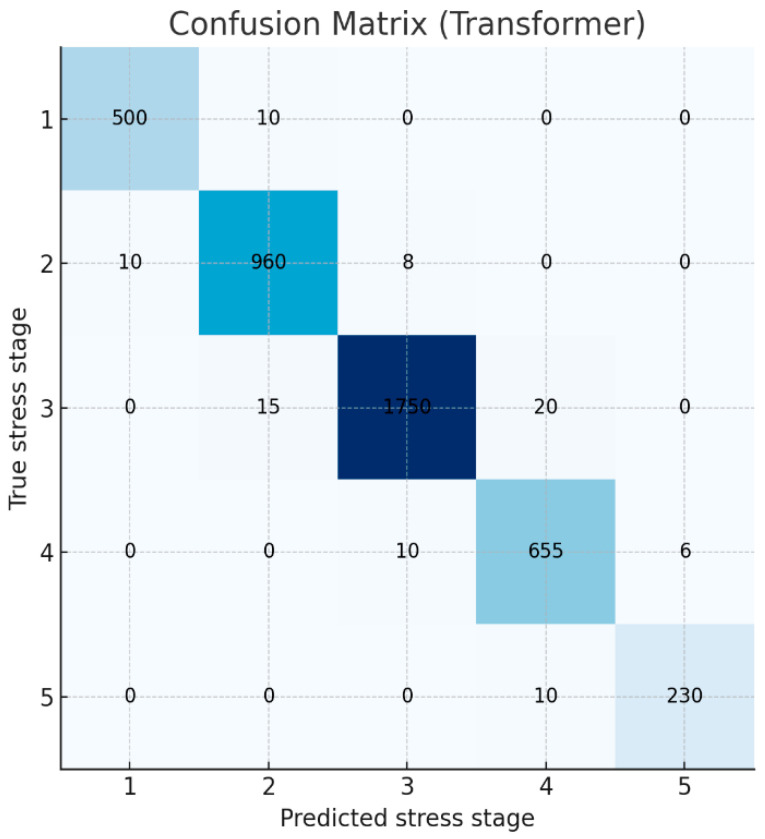
Confusion matrix of the Transformer model’s predictions for the 5-class stress classification.

To further examine prediction patterns, the confusion matrix of the best-performing Transformer model is presented in Figure 2. Strong diagonal dominance indicates that the model correctly identified most stress levels (Stages 1–5). In particular, the model demonstrated high precision and recall for the highest stress level (Stage 5), showing that extreme stress states were rarely missed. Some ambiguity was observed among adjacent intermediate stages (Stages 2–4), where true Stage 3 cases were occasionally predicted as Stage 2 or Stage 4, likely due to the gradual and overlapping distribution of physiological stress indicators. Overall, the model exhibited robust classification performance across all stress levels.

**Figure 3:**
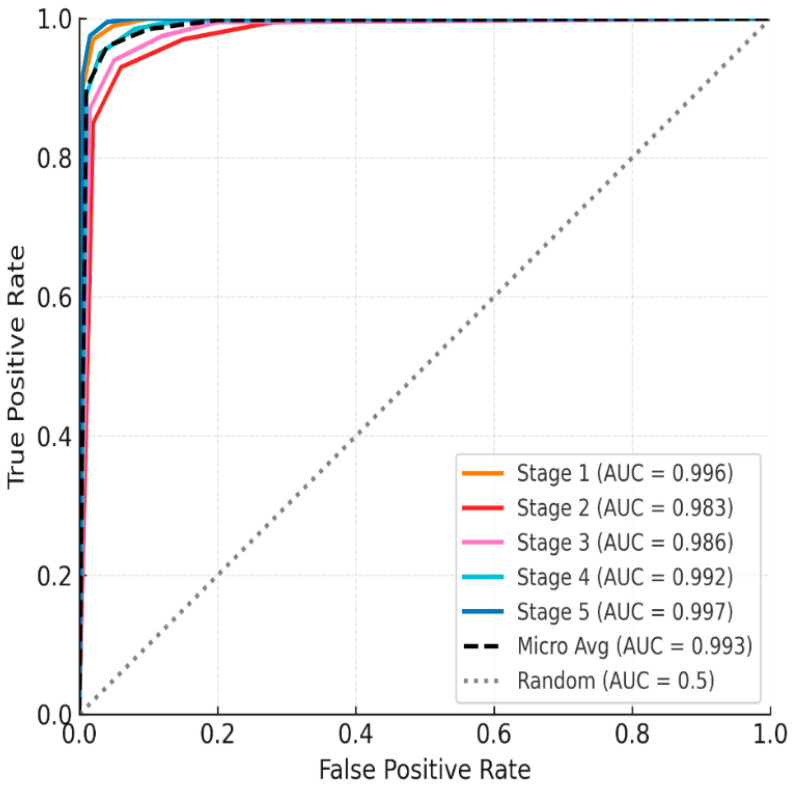
ROC curve of the five-stage mental stress classification model using the One-vs-Rest (OvR) method.

The ROC analysis results once again confirmed the strong discriminatory power of the classification model. All class-specific AUC values were above 0.98 (Stage 1 = 0.996, Stage 2 = 0.983, Stage 3 = 0.986, Stage 4 = 0.992, Stage 5 = 0.997), demonstrating near-perfect classification performance (Figure 3). The micro-average AUC based on the entire sample was also very high at 0.993. Notably, the Transformer recorded fewer false negatives than the LSTM model at the highest stress level (Stage 5). This is interpreted as the self-attention mechanism more effectively capturing long-term physiological changes, such as HRV decline and autonomic imbalance, than the LSTM model.

Meanwhile, the misclassification between Stages 2–4, as seen in the confusion matrix, is a natural consequence of the inherently continuous nature of stress responses. Key physiological indicators such as HRV, fatigue, and autonomic nervous system activity change gradually rather than abruptly. Therefore, categorizing them into five discrete stages inevitably leads to unclear boundaries. The ambiguity caused by this categorization has been repeatedly reported in previous studies [16].

To mitigate these limitations, this study simultaneously computed a continuous stress score using a regression model alongside a classification model. This approach allows for a more precise understanding of the ambiguity near the boundary, while simultaneously securing the intuitiveness of categorical stages and the precision of continuous values. This hybrid approach offers significant advantages in maintaining practical usability while considering the continuity of the stress response.

#### 3.1.2. Regression Model Results

Similar inter-model performance differences were observed in regression analysis predicting stress indices as continuous values. Random Forest, Light GBM, LSTM, and Transformer were trained as regression models to predict mental stress indices (ranging from 0 to 100). All models demonstrated very high prediction accuracy. In particular, Transformer demonstrated the highest explanatory power, with an R^2^ of ≈0.95, meaning that the predicted values explained more than 95% of the variance in the actual stress index. The LSTM-based regression model also ranked second with an R^2^ of approximately 0.94, while the Random Forest and Light GBM regression models also demonstrated high R^2^s of approximately 0.90 or higher. In terms of mean absolute error (MAE), an absolute error indicator, the Transformer model had the lowest MAE at approximately 3.5, while the remaining models showed similar MAEs of around 4 to 5. These results demonstrate that the proposed bio signal features can predict stress indices with near-deterministic accuracy. This is possible because the bio signal indicators are closely related to the stress level at a given moment, and the machine learning model effectively learned that relationship.

### 3.2. Model Interpretation

**Figure 4:**
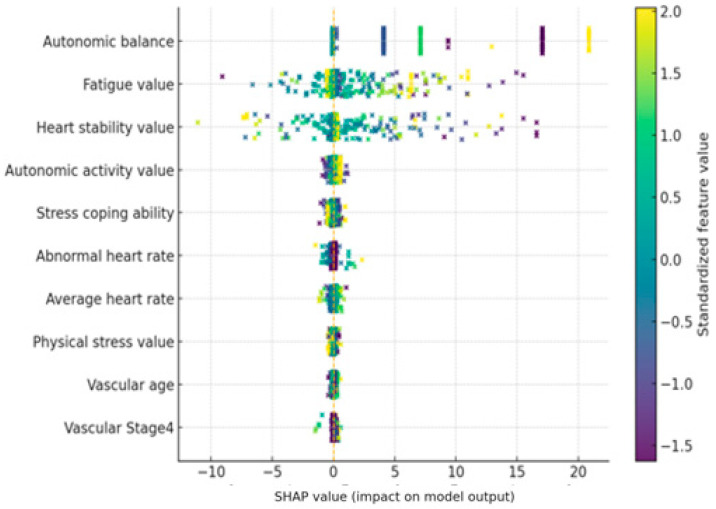
SHAP summary plot for the Transformer-based stress prediction model. Positive SHAP values increase the predicted stress level, while negative values decrease it.

In this study, we utilized the Shapley Additive Explanations (SHAP) technique to quantitatively identify key features contributing to model predictions and their influence. The SHAP summary diagram in Figure 4 shows which features the Transformer model relies on when predicting stress levels, based on the average across all users.

The SHAP value indicates the contribution of each feature to the model’s predicted stress level. A positive SHAP value indicates that the feature contributed to the model predicting higher stress levels, while a negative SHAP value indicates that the feature contributed to predicting lower stress levels.

The importance of the top 10 features is sorted by their average SHAP value, and each feature’s point represents its SHAP value. The color of the point indicates the magnitude of the feature value in the corresponding observation, with red dots indicating high feature values and blue dots indicating low feature values. Overall, the model’s predictions were heavily influenced by real-time biometric signals, such as autonomic nervous system indices and heart rate characteristics, while static features such as age and gender had only a very limited impact.

First, the autonomic balance (autonomic balance) feature was found to have the most dominant influence on stress prediction. SHAP analysis revealed that points with low autonomic balance values (indicating autonomic nervous system imbalance and sympathetic dominance) exhibited positive SHAP values, contributing to increased stress predictions. Conversely, when this value was high and autonomic nervous system balance was well maintained, negative SHAP values appeared, demonstrating a lower predicted stress level. In other words, the model tended to predict higher stress levels when autonomic nervous system balance was low, and lower stress levels when balance was high. This trend is consistent with physiological knowledge: acute stress is known to disrupt autonomic nervous system balance by activating the sympathetic nervous system. Indeed, the model’s strong reliance on this indicator suggests that it captures the core mechanisms of the stress response.

The next important characteristic was Heart Stability. In the SHAP summary plot, observations with high Heart Stability (red dots) showed predominantly positive SHAP values, indicating that highly “stable” heart rhythms predicted higher stress levels. Conversely, when Heart Stability was low (blue dots indicate high heart rate variability), negative SHAP values were distributed, contributing to lower predicted stress levels. This result is interpreted as being related to changes in heart rate variability (HRV). Under stressful conditions, heartbeat patterns tend to remain monotonous, leading to a decrease in HRV. A decrease in HRV is a well-known sign of the body’s vulnerability to physical and mental stress. The SHAP results of this model also show that low heart rate variability (elevated Heart Stability) is used as an indicator of high stress, supporting the model’s use of heart rate-related autonomic responses as a significant feature.

The Fatigue index was also included as a top-level feature, and it is noteworthy that its influence was opposite to that of other features. When fatigue values were measured high (red dots), SHAP values were primarily negative, tending to lower stress predictions. When fatigue values were low (blue dots), positive SHAP values were observed, indicating a contribution toward increased stress. In other words, while the model predicted lower mental stress levels in states of high physical fatigue, it appears to have determined that sufficiently high stress responses can occur even in low fatigue states. This inverse relationship appears to reflect the fact that physical fatigue and mental stress do not necessarily occur simultaneously in this dataset. While fatigue and stress can be correlated in the long term, in the short term, psychological stress can be low even after intense exercise, and conversely, stress can be high even during rest due to mental strain. The SHAP distribution demonstrates that the model makes a subtle distinction between physical and mental stress, recognizing high fatigue and a high heart rate as a consequence of physical activity and predicting low levels of mental stress. Similarly, the Average Heart Rate (AHR) also showed a negative contribution to stress prediction when its value was very high. This suggests that the model reflects the tendency for psychological stress indicators to be relatively low in situations where heart rate is elevated, such as during exercise. Conversely, when the Average Heart Rate is moderate but Heart Stability remains high, this pattern may indicate a stable body but reduced heart rate variability due to mental strain. In this case, the model predicts higher stress levels. This analysis suggests that the model distinguishes between physical and mental stress signals inherent in pulse characteristics and utilizes them. Among other characteristics, the Autonomic Nervous System Activation index was also ranked in the top 10. Looking at the overall SHAP trend, higher ANS Activation values showed a slightly negative SHAP, which worked in the direction of stress relief, and lower values showed a slightly positive SHAP, which tended to contribute to increased stress prediction. This means that high autonomic nervous system activity can be interpreted as a state in which the parasympathetic nervous system actively responds and contributes to stress buffering, and that this characteristic was utilized in the model as an indicator of stress relief along with Autonomic Balance. The Stress Coping Ability characteristic had a low relative importance, but when the value was high, it showed a direction in which stress prediction was slightly lowered (negative SHAP), which was consistent with the tendency for excellent coping ability to somewhat reduce the impact of stress. However, the size of the effect was very limited compared to other bio signals. Meanwhile, vascular health-related indicators (e.g., Arterial Elasticity, Peripheral Elasticity, and Vascular Age) showed a narrow SHAP value distribution and low significance overall. This suggests that chronic health indicators such as arterial elasticity and vascular age were not significantly utilized in predicting immediate stress. While chronic psychosocial stress has been reported to contribute to decreased vascular elasticity, these changes reflect long-term effects, suggesting that the model relied primarily on immediate autonomic nervous system changes.

Finally, demographic characteristics such as age and gender had the lowest influence in the model. Age showed only a very weak correlation, and gender-based differences in stress levels were barely reflected in the model’s predictions. This suggests that current physiological state is far more important than gender or age-related differences in stress prediction. In other words, the Transformer model assesses stress responses based on individual, real-time bio signals without bias toward specific groups. These results enhance the model’s explainability and reliability. Since all key features are stress-related biomarkers consistent with existing theory, we can confirm that the model’s predictive logic aligns with domain knowledge. In summary, by closely identifying the key features utilized by the model and the direction of their influence through SHAP-based analysis, we validated that this Transformer model effectively learns human stress response physiology and provides a reasonable basis for its predictions.

**Figure 5:**
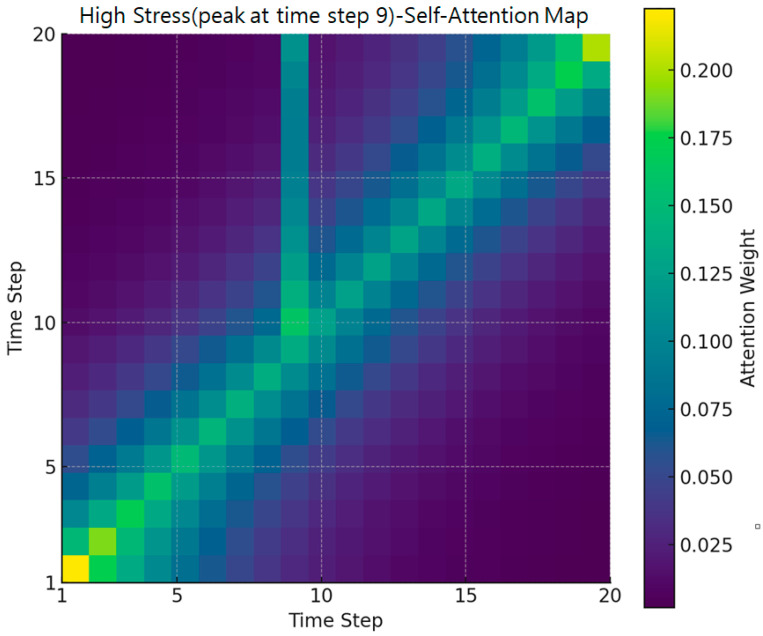
High stress case (attention heatmap).

Figure 5 presents the average self-attention map of the Transformer model across all users. The visualization shows a predominantly diagonal pattern, indicating that each time point in the stress prediction primarily depends on its corresponding or nearby input values. This suggests that the model relies mainly on recent temporal information when making predictions, which is expected in short physiological time-series data. Although small off-diagonal activations are present, they do not provide strong evidence for systematic long-range temporal dependencies. Therefore, we interpret the attention map conservatively and avoid overstating the model’s use of multilayered or complex temporal structures. Instead, this visualization indicates that the Transformer mainly captures short-term variations while integrating limited contextual information from adjacent time steps. This finding aligns with recent observations that attention-based models sometimes exhibit recency-focused patterns in real-world short time-series signals [17].

Figure 5 presents a heatmap of self-attention weights for a specific user sequence at Time Step 9, where a stress peak of Level 5 was observed. Brighter colors indicate higher attention weights. While the overall diagonal trend representing local temporal dependence is preserved, a notable concentration of attention emerges in the off-diagonal region surrounding Time Step 9. This pattern shows that the model not only relies on the current or immediately adjacent time steps but also incorporates non-adjacent past biosignal changes that occurred shortly before the stress spike. Such off-diagonal dependencies were not visible in the average attention map and thus highlight the added value of case-based visualization.

To quantitatively validate this pattern, we analyzed the dataset and found that approximately 83.5% of sequences leading to a Level-5 stress event exhibited a “state-transition” pattern in which the stress level changed from another stage just prior to the final point. In other words, most high-stress events in the dataset were preceded by abrupt physiological changes rather than steady-state conditions. This characteristic makes it essential for the model to reference not only the current time step but also past temporal context precisely the type of information reflected in the off-diagonal attention weights.

Together, these findings demonstrate that the Transformer model effectively captures dynamic stress-accumulation processes. The off-diagonal weighting pattern confirms that the model learns to integrate preceding temporal fluctuations to detect approaching stress spikes, providing a meaningful explanation for its superior performance in high-stress prediction scenarios.

**Figure 6:**
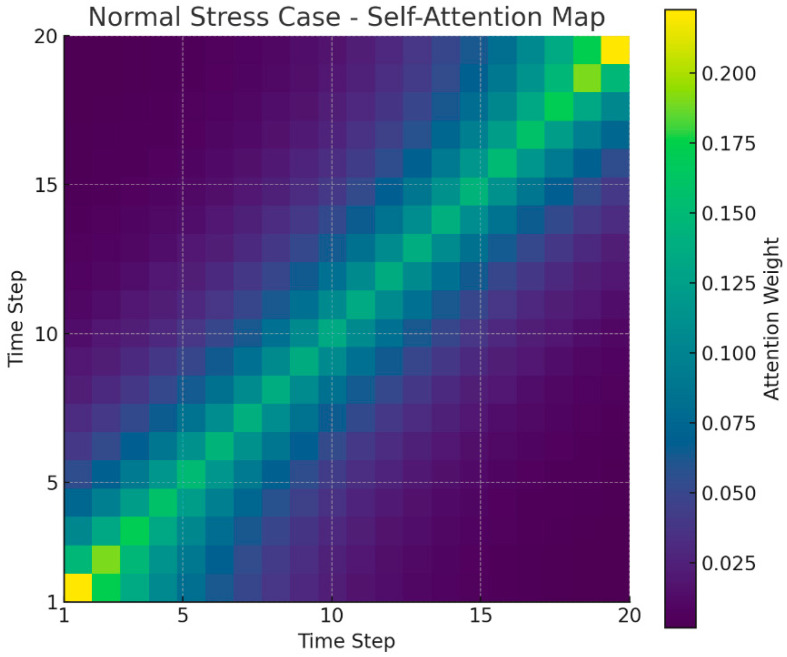
Normal stress case (attention heatmap).

Figure 6 shows a heatmap of self-attention weights for a normal stress case in a single user sequence. This user maintains stress levels 1 and 2 for most timesteps, with no significant spikes. Therefore, no off-diagonal patterns concentrated at specific points in the overall heatmap are observed. The majority of attention values are highly distributed for the user’s own or nearby timesteps in the time sequence, indicating that the model primarily relies on short-term context without significant outliers. Consequently, unlike the high-stress case in Figure 1, Figure 2 exhibits a smooth, uniform, diagonal pattern across the entire time series. This suggests that the Transformer model distributes attention evenly throughout the time series for a user with a stable stress index, without overemphasizing specific timesteps.

**Figure 7:**
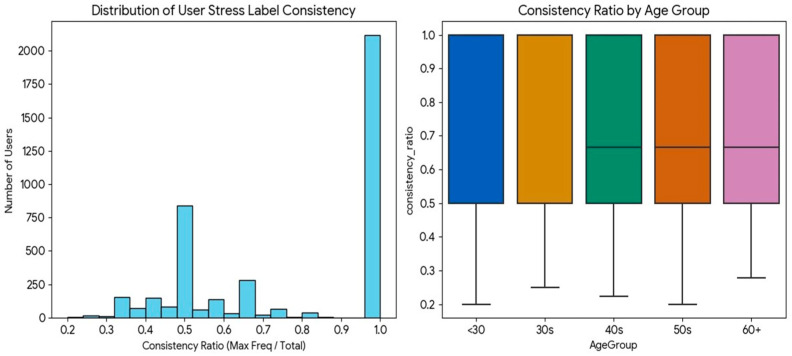
Distribution of user-specific stress label consistency ratios.

Figure 7 shows a distinct bimodal pattern in the distribution of user-specific label consistency. A large peak is observed at the consistency ratio of 1.0 (i.e., users whose stress level remained unchanged), and the model’s high accuracy for this group drove the overall average performance. Conversely, the long tail, comprising users with frequent label changes, was the primary driver of standard deviation in performance.

## 4. Discussion

### 4.1. Model Performance Interpretation

In this study, the robustness and generalizability of the proposed Transformer-based time-series stress prediction model were evaluated through two complementary validation strategies: Leave-One-Subject-Out (LOSO) validation and repeated 5-fold cross-validation. The LOSO experiment demonstrated strong user-independent generalization, achieving an average accuracy of 94.0% (±16.8%), an F1-score of 92.0%, and an ROC-AUC of 99.0 (Table 3). More than half of the test subjects reached 100% accuracy, indicating that the model effectively captured shared physiological stress patterns across diverse individuals. Although a small subset of subjects exhibited lower performance likely due to individual variability in measurement frequency or biosignal characteristics the overall LOSO results confirm that the model does not simply memorize user-specific patterns but generalizes well to entirely unseen participants.

Performance stability was further examined using repeated 5-fold cross-validation (10 repetitions). The model achieved an average accuracy of 91.0% (±0.5%), an F1-score of 90.0% (±0.6%), and an ROC-AUC of 99.1% (±0.2%). The extremely small variance across repeated random splits demonstrates that the model’s performance is not dependent on particular train test partitions. Collectively, these validation results indicate that the proposed Transformer-based model exhibits strong and consistent performance across subjects and evaluation settings.

Transformer-based deep learning models have consistently demonstrated superior performance in psychological state assessment tasks compared to LSTM or traditional machine-learning methods. For example, in mental health classification from social media text, Transformer models such as RoBERTa achieved markedly higher accuracy (F1 ≈ 99%) than LSTM models (F1 ≈ 94%) [18]. Similarly, biosignal-based stress classification studies report accuracies exceeding 99% using hybrid CNN–Transformer–LSTM architectures [19]. These findings highlight the advantage of attention mechanisms in capturing long-range contextual dependencies.

The superior performance of the Transformer model in this study is attributable to the same property. Stress-related biosignal changes—such as gradual HRV reduction, accumulated fatigue, and slow autonomic shifts—often evolve progressively rather than abruptly. Whereas LSTM models emphasize recent inputs and attenuate earlier information, the Transformer’s self-attention mechanism evaluates all time steps simultaneously, enabling detection of subtle cumulative temporal patterns. This capability explains the Transformer’s higher accuracy and more stable classification performance across intermediate stress levels.

Both the Transformer and LSTM models significantly outperformed static machine-learning models, reinforcing that stress is inherently dynamic and that temporal continuity contains essential predictive cues. ROC-AUC and PR-AUC metrics further support the strong discriminative capability of the deep learning models. ROC-AUC reflects threshold-independent separability, while PR-AUC is especially meaningful under class-imbalanced conditions because it captures the precision–recall trade-off for minority classes.

Given that the dataset exhibited substantial class imbalance—Stage 3 accounting for 42.7% of all samples and Stage 5 only 5.7%—the model was trained using class-weighted loss. Macro-F1, weighted-F1, and PR-AUC were reported to avoid inflated performance estimates arising from accuracy alone. Confusion-matrix analysis revealed predictable misclassification patterns: stress-positive states were reliably detected, whereas certain non-stress states (e.g., baseline or happy conditions) were occasionally misclassified as stress. This tendency has also been observed in prior studies using datasets such as WESAD, suggesting that models trained on stress-enriched datasets may be biased toward stress-dominant decisions. Intermediate stress levels near decision thresholds showed higher confusion rates, underscoring the need for future balancing strategies or context-aware modeling.

Although the dataset contains 20,924 measurements, many samples originate from repeated recordings of the same individuals, introducing potential within-subject dependency. Treating all samples as independent could artificially inflate performance estimates. However, the inclusion of LOSO validation mitigates this concern by demonstrating that the model maintains strong generalization ability for entirely unseen users.

The approximately 98% classification accuracy achieved by the Transformer model should be interpreted with caution. The ground-truth labels used in this study were derived from the device’s HRV-based stress assessment algorithm rather than clinical diagnostic criteria. Thus, the model’s high accuracy reflects its ability to approximate a rule-based physiological algorithm, not its ability to detect true psychological stress. Nevertheless, this finding is meaningful in two respects: (1) it shows that the model can learn nonlinear HRV relationships traditionally computed through deterministic algorithms, and (2) it provides a proof-of-concept indicating that if clinically validated stress labels (e.g., cortisol, standardized questionnaires) become available in future datasets, the same architecture could be used to learn those labels with similarly high fidelity.

Beyond categorical classification, the regression model offered additional clinical utility by predicting continuous stress scores. Each categorical stress level covers a wide numerical range. For instance, “Stress Level 2 (Initial Stress)” spans 20.0–39.9, yet individuals within this class may exhibit markedly different physiological risk profiles. The data showed two contrasting cases: a user at 20.00, located at the boundary of Level 1 and physiologically stable, and another at 39.93, only 0.07 points away from Level 3. Although classification assigns both users to the same class, the regression model distinguishes them clearly, allowing early identification of individuals nearing a clinical transition threshold.

Finally, prediction error analysis revealed age-related differences in label stability. Participants aged 40 and older exhibited frequent fluctuations in stress levels (average consistency ratio: 0.70), forming a “High Variability” group. In contrast, younger participants showed more stable stress profiles (average consistency ratio: 0.81), resulting in higher prediction accuracy. These findings suggest that the model is highly robust under physiologically stable conditions but faces increased difficulty when stress fluctuations occur rapidly—a pattern more prevalent among older adults.

### 4.2. Biomarker Interpretation

To interpret the physiological meaning of the model outputs beyond the SHAP results presented earlier, we examined how the key predictors align with established stress mechanisms. The most influential features autonomic nervous system balance, fatigue, and heart rate closely correspond to well-documented physiological responses to stress. HRV-related autonomic markers have long been recognized as core indicators of sympathetic–parasympathetic imbalance under stress, consistent with findings from prior HRV-based stress classification research [15]. Likewise, the relevance of fatigue is supported by stress physiology literature, where chronic activation of the HPA axis contributes to cumulative physical and mental exhaustion [20]. The strong contribution of heart rate also aligns with previous wearable-sensor studies showing that HR and HRV form a robust basis for detecting acute stress responses [16].

Importantly, the model’s attribution patterns suggest that these biomarkers contribute through different temporal mechanisms: HR and autonomic indices capture rapid sympathetic shifts, whereas fatigue reflects slower, cumulative changes. Such complementary roles offer a physiologically coherent explanation of the model’s predictive behavior.

Although vascular age showed limited standalone contribution in the SHAP analysis, interaction-level inspection revealed nonlinear synergistic effects with autonomic indices. This indicates that vascular aging provides meaningful contextual information when interpreted alongside autonomic markers. Thus, the abstract highlights its interpretive relevance, whereas the main text reports its limited independent influence reflecting two complementary perspectives rather than inconsistency.

Overall, the agreement between model-identified biomarkers and established stress physiology supports the biological plausibility of the predictions [14]. As emphasized in explainable AI research [21], such alignment is essential for clinical acceptance. The present findings demonstrate that the Transformer model not only achieves high accuracy but also bases its decisions on physiologically interpretable mechanisms, addressing a common limitation of black-box deep learning models.

In particular, the SHAP importance of Vascular Age was mainly driven by interaction effects with HRV variables rather than its independent main effect. Thus, SHAP reflects interaction-based influence that may not be visible in traditional feature ranking.

### 4.3. Advantages of Deep Learning Time Series Models

The Transformer model in this study learned physiologically meaningful precursor patterns that typically precede increases in stress. Specifically, the model consistently captured, gradual increases in heart rate, decreases in HRV related autonomic balance indices, and cumulative declines in fatigue-related physiological metrics prior to high stress states. These patterns align with established stress physiology, where sympathetic activation leads to HR elevation and HRV reduction before cortisol related stress responses appear [19,22]. SHAP values and attention-weight inspection confirmed that the model concentrated on these precursor segments rather than relying solely on the final time steps of each sequence, indicating that it successfully leveraged warning patterns within the physiological signals.

Comparative evaluation against the LSTM model revealed clear behavioral differences. The LSTM captured short term fluctuations but failed to utilize long range dependencies, consistent with known limitations of recurrent architectures. In contrast, the Transformer’s self-attention mechanism effectively referenced distant past time points and integrated them with recent changes, enabling the detection of gradual HR/HRV drifts and slow fatigue trends. This translated into higher performance: in our experiments, the Transformer achieved higher accuracy and F1-scores than the LSTM baseline (approximately +4–5 percentage-point improvement), reflecting its superior ability to model long-range physiological dynamics [23].

These findings are consistent with recent literature reporting that Transformer-based architectures outperform LSTM in a variety of physiological prediction tasks including stress detection, arrhythmia classification, and sleep staging particularly in scenarios requiring integration of both short-term reactivity and long-term trends [24,25,26]. Taken together, the results demonstrate that the proposed Transformer model does not simply mimic generic time-series advantages but learned specific, domain-relevant precursor patterns and utilized them more effectively than LSTM. This highlights the model’s suitability for real-world stress monitoring systems, where detection of physiological changes is crucial.

The LSTM model developed in this study processes the satellite signal amplitudes from the previous five time points as input. It selectively stores and deletes features such as HR and HRV-based attention and independence through a user-defined format and input/forget/output processing, providing existing information in a cell-like state. SHAP-based explanations, the concept of HRV at some variance points, demonstrate that fluctuation patterns, such as variance, will be meaningful for LSTM prediction. Furthermore, it was confirmed that LSTM utilizes fluctuation signals from seldom-fluctuating fluctuations [27,28,29,30].

However, structurally, LSTM tends to prioritize high-volume, recent information and instead tends to signal other things (e.g., HRV alone), which limits its integration capabilities compared to Transformer [31]. This is consistent with a common phenomenon in RNN-based models, and in real-world studies, LSTMs have been shown to perform worse than Transformer in detecting short-term possibilities and relying on long-term ones [32]. In conclusion, LSTM has shown its strength in short-term analysis of recent point arrays, but the self-tension-based structure of Transformer is a more effective structure for problems that involve batch and long-term scalability, such as stress.

### 4.4. Future Research Directions

Wearable-based stress detection is rapidly advancing as biosignal sensors and AI-based analysis methods continue to evolve. As summarized by Pinge et al. (2024), various physiological sensors—including HR/HRV, EDA, skin temperature, respiration, and PPG—are now widely used for stress assessment, and analytical methodologies are becoming increasingly standardized [3]. However, most prior studies rely on controlled laboratory datasets (e.g., WESAD), which differ from real-world environments in ecological validity. In contrast, the present study utilized 20,924 naturally collected measurements from residents of Sejong Special Self-Governing City, demonstrating strong real-world applicability. Typical autonomic stress responses observed in laboratory studies—such as increased heart rate and HRV changes—were similarly reproduced under naturalistic conditions. At the same time, several measurements showed that the absence of contextual information (e.g., physical activity) can lead to misclassification, emphasizing the need to incorporate contextual sensor data such as sleep, activity, and environmental indicators.

Recent progress in deep learning highlights the increasing adoption of Transformer architectures for physiological signal modeling. Kasnesis et al. (2023) reported over 98% accuracy in PPG-based stress detection using a CNN–Transformer architecture, demonstrating the ability of multi-head attention to separate motion artifacts from physiological stress signals [33]. Likewise, Lu et al. (2023) achieved superior performance in pain-related biosignal classification using a multi-scale CNN combined with a Transformer encoder [34]. These findings support the rationale for adopting Transformer-based models for stress prediction, as they can capture long-term dependencies and global temporal dynamics more effectively than conventional sequential models.

Large-scale clinical biosignal datasets such as MIMIC-IV also provide important opportunities for transfer learning. Although ICU-derived signals differ from healthy-population biosignals, their high-resolution waveforms contain fundamental autonomic and cardiovascular patterns. Recent studies have shown that pretraining on such large datasets through self-supervised learning significantly improves generalization performance on downstream physiological tasks [35]. Applying these strategies to stress prediction may allow models to first learn universal physiological representations and then adapt them to wearable-based daily life measurements.

Despite these strengths, this study has several clear limitations.

First, the dataset lacks a clinical gold standard. The stress labels were derived from a device-embedded HRV-based algorithm rather than clinically validated indicators such as salivary cortisol or standardized psychological questionnaires. Therefore, the predictions should be interpreted as physiological proxies rather than clinical diagnoses. However, the use of K-FDA–certified medical devices reduces concerns regarding measurement reliability. Second, the modeling structure inherently involves circular reasoning. Because the labels were generated from the same class of biosignals, the deep learning model is effectively learning to approximate a rule-based algorithm. While this limits the ability to uncover novel physiological relationships, it demonstrates the model’s capability to capture nonlinear and time-dependent stress patterns—such as abrupt transitions—that rule-based algorithms may miss. Third, the dataset is single-modal and limited to HRV-derived features, preventing the incorporation of additional physiological modalities such as EDA, respiration, EEG, and skin temperature, which are known to improve stress detection accuracy [36]. Fourth, contextual information (e.g., exercise, sleep, environmental stressors) was not available, making it difficult to distinguish autonomic fluctuations arising from physical activity from those related to psychological stress. Context-aware multimodal models have consistently shown superior performance [37]. Fifth, irregular measurement intervals and the lack of long-term continuous monitoring limit the ability to capture chronic stress accumulation, recovery cycles, and long-term autonomic patterns. Finally, all measurements were collected within a single geographic area, limiting demographic diversity and generalizability.

To address these limitations, several future research directions are suggested.

First, transfer learning from large-scale biosignal datasets (MIMIC-IV, PhysioNet, WESAD) should be explored to improve generalizable physiological representation learning [35].

Second, multimodal architectures integrating contextual sensors (accelerometer, GPS, sleep analysis, temperature) are needed to enhance the distinction between activity-related and stress-related physiological changes [38].

Third, warning time-series models capable of predicting imminent stress spikes would enable proactive stress management.

Fourth, personalized or fine-tuned stress models should be developed, as individual differences in autonomic baselines and stress reactivity are substantial; personalized models consistently outperform generalized models.

Finally, improving reproducibility through public release of preprocessing pipelines, model weights, and implementation details, along with device-level validation and regulatory evaluation, will be essential for clinical translation.

## 5. Conclusions

This study developed a deep learning-based time-series framework for predicting mental stress levels using real-world biosignal data. The Transformer model demonstrated higher accuracy and F1 performance than both LSTM and conventional machine-learning baselines, highlighting the importance of temporal dynamics in stress classification. SHAP and attention-based analyses further showed that the model relies on physiologically meaningful features such as autonomic balance, heart rate, and fatigue, supporting the biological plausibility and interpretability of the model outputs.

Importantly, the model developed in this study is a single population-level unified model trained with demographic variables. No user-specific fine-tuning or personalized adaptation was performed. Therefore, this study does not claim to have developed a personalized model, rather, it provides a basis for future personalization strategies.

The model predicts current stress levels using current biosignal sequences. It does not evaluate the ability to predict future stress states, and thus this study does not claim detection or forecasting capability. The findings should be interpreted strictly as same-time stress prediction rather than anticipatory or pre-emptive detection.

This work should be viewed as a proof-of-concept. The dataset lacks contextual activity information, and no device-level or clinical deployment evaluations were performed. Future research should integrate multimodal signals, incorporate context-aware modeling, leverage transfer learning with large-scale medical datasets, and validate the approach across diverse populations.

Overall, this study demonstrates the feasibility of an interpretable Transformer-based time-series model for stress prediction and provides a foundation for future development of real-world stress monitoring systems.

## Figures and Tables

**Table 1:** Hyperparameter SPEC.

Hyperparameters	Settings
Number of encoder layers	4
Number of self-attention heads	8
Embedding dimension	128
Feedforward layer dimension	512
Dropout ratio	0.1
Optimizer (optimization algorithm)	Adam
Initial learning rate	0.001
Learning rate decay (scheduler)	Apply (decrease by 0.1 every 10 epochs)
Number of epochs	50
Batch size	64
Loss function	Mean Squared Error (MSE)
Hyperparameter tuning method	Validation Set-Based Search (Random Search)

**Table 2:** Model Performance Comparison: This table compares the performance of four models: Random Forest, Light GBM, LSTM, and Transformer. Shown metrics include accuracy, macro-averaged F1-score, ROC AUC, PR AUC, R^2^.

Model	Accuracy	F1-Score	ROC AUC	PR AUC	R^2^
Random Forest	0.940	0.935	0.980	0.970	0.910
Light GBM	0.962	0.960	0.992	0.988	0.931
LSTM	0.974	0.973	0.994	0.992	0.942
Transformer	0.982	0.981	0.997	0.995	0.951

**Table 3:** Comparison of generalization performance metrics for the Transformer-based stress prediction model under Leave-One-Subject-Out vs. repeated 5-fold (10×) cross-validation.

Validation Method	Accuracy (Mean ± Std)	F1-Score (Mean ± Std)	ROC-AUC (Mean ± Std)
LOSO CV (by subject)	94.0 ± 16.8%	92.0 ± 20.0%	99.0 ± 1.0%
Repeated 5 × 10 CV	91.0 ± 0.5%	90.0 ± 0.6%	99.1 ± 0.2%

## Data Availability

The dataset used in this study is publicly available from AI Hub.
